# Some Comparative Qualities of Oxyphosphate and Silicate Cements

**Published:** 1908-06-15

**Authors:** R. B. Tuller


### SOME COMPARATIVE QUALITIES OF OXYPHOSPHATE AND SILICATE CEMENTS.

By R. B. Tuller, D.D.S.

Users of cement fillings of any considerable experience know that the oxyphosphate cements cling with some degree of permanency to the walls of the tooth cavity. The wasting or wearing away is usually in the middle, and that next to the walls is the last to give way. We find many such fillings hollowed out extensively in the middle, but still covering most of the walls, so that the tooth is still well preserved.

Operators, know, too, as far as their experience has gone with the superior grades of silicate cements, and also from the instructions given by the producers, that a good retentive cavity hold is a necessity to this class of cement, or so-called enamel filling; and while the silicates are apparently the stickiest kind of cements in their plastic state, when they become crystallized they do not, in the mouth, seem to retain their tenacity to the walls as do the oxyphosphates. That is to say, an oxyphosphate filling may be depended upon to do all that we can expect of this material generally, in cavities that are not undercut, or made specially retentive, while it is not so of the silicate cements. Usually, however, where we do use them, the cavity is naturally retentive, but we do put them sometimes in places that are not retentive—that have diverging walls—and expect them to stay. In fact all inlay work that depends on cement calls for diverging walls. If we were to use a silicate cement to set an inlay, and it may be done, both the cavity wall, after the matrix has been fitted or the impression taken, and the inlay should be undercut.

Now, it has been claimed, and the authority is not questioned, that laboratory tests of the silicate cements show a decided tendency to shrink; they do shrink, of course, when they will rattle around or easily slide out of the glass tube in which the test is made. The maker of cements, or some of them, say they do not care what these tests show out of the mouth; that in the mouth they do not shrink from the walls to fhe cavity. My own experience has been entirely with fillings in actual practice in the mouth, and where the cavity is made retentive I have never seen, in the many that I have made, a single one that showed a line of separation from the margins, if proper attention has been given to forcing the material to the walls and margins at the time of insertion. In a few instances in my early experience I have had inlays come out very soon after they were set, and one or two fillings came out that were inserted in cavities with diverging walls without any undercuts.

This leads me to conclude that when the material is retained properly until it has crystallized, that such shrinkage as takes place, admitting that there may be some in the mouth, is toward the cavity walls or margins, and not away from them, for as before stated I have never seen in any filling properly held in place, the minutest degree of separation from the margins. Those that were not held by some anchorage seemed to cleave from the cavity contact. Of course a cavity of retentive shape is preferable with oxyphosphate cement, but rarely if ever does a properly mixed and manipulated filling of such material cleave wholly from the cavity walls.

No doubt, moisture has some influence as regards this cleavage, and for one thing it comes in contact with the filling before it is thoroughly crystallized. In regard to the enamel fillings, they must be kept absolutely from contact with water from fifteen minutes to half an hour, and longer, the longer the better; while with the oxyphosphates the advice is with some of them, called hydraulic or submarine, to wet them or allow the saliva to come in contact shortly after insertion. As to clinging qualities, it is well known that the portion of either of these cements that hardens out of the mouth on the slab and spatula free from moisture may be the more readily removed from either by wetting. The oxyphosphates will usually scale off in pieces of varying size, leaving the slab quite clean, while the enamel will be much harder to remove, notwithstanding what has been said about cleaving from the tooth cavity walls when not duly anchored. It seems almost a contradictory statement of cement qualities, but it must be taken into account that the mixing slab is usually polished glass or porcelain, and the spatula more so perhaps, even of bone, than the walls of the cavity; and again, the hardening under dry conditions. In one case moisture is advised, or not specially guarded against, after a few moments, while the contrary is imperative of the other for considerable time—the longer the better. Any one who has undertaken to remove the enamel from a spatula that has hardened in a dry environment for hours knows that it clings to the bone spatula as though a part of the bone, and though it may be removed easier if dipped in water it takes considerable effort to remove it all. If our tooth cavity was thoroughly dehydrated with absolute alcohol or chloroform after wiping dry, and could be kept dry, including the enamel filling, for hours, instead of minutes, we might, and no doubt would, get better results than we comprehend, doing as we do. We coat them with varnish or melted paraffine. How perfectly that protects and how long is uncertain. If varnish is too thick it takes a long time to dry, and it does not get a hold on the filling as does very thin varnish, and the thin dries very quickly. I imagine if varnish has dried only to a tacky consistency, as does a thick varnish within any reasonable time, it is largely removed with the rubber dam, and in that tacky condition what remains is easily attacked by moisture, destroying its efficiency as an impervious coating. Paraffine to get any tenacious hold, if it does at all must be made pretty hot—hotter, perhaps, than the sensitive tooth will stand. In using the latter it is my habit to flow plentifully in case of proximal fillings, filling the space between the teeth and then cutting my dam away instead of dragging it off the teeth to bring the paraffine with it. It would, perhaps, be better to cut it off in case of a varnish coating.

Of course, as a rule, the dentist has to pursue a course consistent with the time allotted for the work in hand, and the patient's convenience and comfort has to be considered. The rubber dam might be left on for an hour or more in some particular case, and with certain patients, or the dam may be cut away, leaving a suitable portion around the tooth to be brought down, gathered and tied, but such things, while desirable, perhaps, are not generally practicable. However, some means that is reasonably practicable might be devised that would keep enamel fillings dry for a much longer time than we do, with a certainty of better results. In time, perhaps, we may have a submarine enamel, since the liquid used in both classes of cements is practically the same—that is, some form of phosphoric acid.

The silicate cements that I have used in my practical experience are Ascher's, for about three years, and Translux (Caulk's), for a few months. In the mix and manipulation I can see no difference. Of the constituent parts of either I know nothing, except the claims of the producers. I find both changing in shade a few points after a short time (over night), and in consequence, as the shade grows lighter, I aim to mix a shade deeper than I want, to allow for this sort of bleaching out. With all that, there is no time that the filling looks so well so in harmony with the tooth as when it is first done, and before removal of the dam. And yet, taking all in all, the enamel fillings are, in my estimation, a nearer approach to the ideal, esthetically considered, at least, and taking into account the *average*, and not the high, artistic attainments of experts in poreclain inlay work. The ditch around most of the average porcelain inlays, some time in use, filled usually with a dark debris, though ever so shallow, marks the outline of the inlay as plainly as an old crack in a porcelain dinner plate, and the harmony of shade in much of this work is not so good as an artistic eye can get with enamel filling; and this I say as still an enthusiast in porcelain inlay work considerately placed.

My experience with the enamel fillings has been to put them to test in almost all places, and all teeth in the mouth where I might use any other filling material, and generally the result has been highly satisfactory to me and my patients. The failures are mostly my own fault in mix or manipulation, and one commendable thing with such cases, they are soon apparent and easily corrected. Some faults in shade—a misjudgment in selection of powder or in mixing two colors, is often easily corrected by simply cutting out the exposed portion that offends, and, with new anchorage or retention secured in what remains, a new facing is put in, saving much time and trouble. Other defects may be remedied the same way. The only thing to guard particularly against is not to have the joining of the old and new exposed to view, for even with the same powder the joining can be detected. If this came along some part of the labial exposure it would be noticeable.

I have some rather extensive contours built out with enamel in the anterior teeth, and while they will not stand everything without showing wear, the patients wearing them prefer to have them renewed from time to time to having anything else. In occluso-proximal cavities in both molars and bicuspids they stand up surprisingly well under the usual wear and tear of mastication. For small pits and fissure fillings the enamel is par excellence, and perhaps where one would hesitate as much as any place, I have found them remarkably adapted to cervical channels, that follow the gum line in many cases. I simply remove the decay, define the margins with healthy enamel, annd unless decay requires it, do not go deeper, possibly, than the thickness of the enamel, and then slightly undercut the entire length and at ends of the cavity with either small inverted cones or suitable wheel burrs. Such fillings are the least disfiguring, even if the shade is not perfect, of anything I know, not excepting porcelain, which would be impractical anyway in such proportioned cavities as the narrow curved lines I refer to. Of course, it is equally as satisfactory in cavities of larger proportions. If such cavities go a whole year, and two years, without a sign of failure, can we not feel some confidence in their continued integrity?

Regarding the selection of shades, the guides, so far as I have seen (if any at all), with the powder, are of little value. One should either mix a little from each bottle, shape as desired, and press on to polished glass to harden; or mix a little powder with water as thick as it will drop or flow, and either put a little in the cavity prepared or drop a bit on the surface of the tooth to note the harmony. Or, two colors or more, may be mixed up the same way to see how close a match can be got, favoring a shade of the material a trifle darker than the tooth, for it will bleach out some, as before stated.

If little pellets are made in the regular way, from each bottle, and then an exact half and half from each bottle with every other color, one would have, with a box of half a dozen shades, no less than twenty-one exact shades that may be produced at will and be used to more intelligently make other variations. These guide buttons or pellets would have to be marked with the shade numbers used to produce them.

There are places and conditions where the oxyphosphate cements may be used as a foundation for the enamel, or rather the heart of the filling, with a heavy veneer of enamel. In other words, *oxyphosphate dentine* and *silicate enamel*, the latter, however, being heavier than nature's enamel, and the mixing should be done so that the enamel could be spread on the yet somewhat fresh cement, with a view to having them perfect a union. If the oxyphosphate cement is allowed to harden before the enamel is put on, then anchorage or retention should be provided.—*American Journal.*

